# Epigenetic regulation of cancer stem cell formation and maintenance

**DOI:** 10.1002/ijc.33398

**Published:** 2020-11-30

**Authors:** Rhiannon French, Siim Pauklin

**Affiliations:** ^1^ Botnar Research Centre, Nuffield Department of Orthopaedics, Rheumatology and Musculoskeletal Sciences University of Oxford Oxford UK

**Keywords:** cancer stem cells, cancer therapy, early detection of cancer, epigenetics, induced pluripotent stem cells, tumorigenesis

## Abstract

Cancerous tumours contain a rare subset of cells with stem‐like properties that are termed cancer stem cells (CSCs). CSCs are defined by their ability to divide both symmetrically and asymmetrically, to initiate new tumour growth and to tolerate the foreign niches required for metastatic dissemination. Accumulating evidence suggests that tumours arise from cells with stem‐like properties, the generation of CSCs is therefore likely to be an initiatory event in carcinogenesis. Furthermore, CSCs in established tumours exist in a dynamic and plastic state, with nonstem tumour cells thought to be capable of de‐differentiation to CSCs. The regulation of the CSC state both during tumour initiation and within established tumours is a desirable therapeutic target and is mediated by epigenetic factors. In this review, we will explore the epigenetic parallels between induced pluripotency and the generation of CSCs, and discuss how the epigenetic regulation of CSCs opens up novel opportunities for therapeutic intervention.

**Abbreviations**
AML: acute myeloid leukaemia
ATP: adenosine triphosphate
BAF: BRG1‐ or BRM‐associated factors
BER: base‐excision repair
BET: bromodomain and extra terminal
BRD: bromodomain
CIMP: CpG island methylator phenotype
CSC: cancer stem cell
DNA: deoxyribose nucleic acid
DNMT: DNA methyltransferase
EMT: epithelial‐mesenchymal transition
ESC: embryonic stem cell
FACs: fluorescence‐activated cell sorting
GBM: glioblastoma multiforme
HDAC: histone deacetylase
IL6/17: interleukin 6/17
iPSC: induced pluripotent stem cell
lncRNA: long noncoding RNA
LSD1: lysine‐specific demethylase 1
MET: mesenchymal‐epithelial transition
miRNA/miR: microRNA
ncRNA: noncoding RNA
PDAC: pancreatic ductal adenocarcinoma
PRC: polycomb repressive complex
RISC: RNA‐induced silencing complex
RNA: ribose nucleic acid
SAM: S‐adenosyl methionine
SWI/SNF: SWItch/sucrose nonfermentable
TAM: tumour‐associated macrophage
TET: ten‐eleven transcription factor
TGF: transforming growth factor
TME: tumour microenvironment
VEGF: vascular endothelial growth factor

## 1 INTRODUCTION

As with adult tissues, cancerous tumours also contain a rare subset of cells with stem‐like properties that can function to regenerate the heterogeneous cell populations observed therein. These cancer stem cells (CSCs) are defined by their ability to divide both symmetrically and asymmetrically, to initiate new tumour growth and to tolerate the foreign niches required for metastatic dissemination. As the tumour‐initiating population, CSCs underpin the very nature of malignancy and studying their regulation is essential for understanding tumour formation, metastasis and relapse after therapy.

As it is not possible to isolate CSCs based on functional properties, CSC identification can be achieved by FACs sorting based on surrogate cell surface marker profiles and subsequent transplantation into immune‐compromised mice to demonstrate enhanced tumourigenic potential. Using this strategy, CSCs have been identified in most cancers, first in acute myeloid leukaemia (AML) followed by breast cancer and other solid malignancies such as brain, colon and pancreatic cancer, and are purported to account for only a few per cent of the total cell population.^1^, ^2^, ^3^, ^4^, ^5^, ^6^ The existence of a rare population of CSCs supports the notion that a heterogeneous cancer arises from a single cell atop a cellular hierarchy. This also suggests that cancer arises from a cell with stem‐like properties, as the cell‐of‐origin would require the asymmetric division to initiate and maintain tumour growth. Furthermore, the existing properties and long lifespan of a stem cell make it more likely than a differentiated cell to acquire a tumourigenic phenotype.^7^ Recent evidence supports the premise that cancer arises from the deregulation of existing stem cell populations. In an organ‐wide study, Zhu et al induced oncogenic mutations specifically in CD133+ cells in the mouse. Tumours only arose in those organs where CD133 was proven to have generative capacity, that is, was an effective marker of a normal stem cell population (the liver, small intestine and stomach but not brain, kidney or pancreas). Furthermore, liver injury increased CD133+ cells and tumourigenic potential after transformation, thus indicating environmental factors can converge with genetic mutations to increase cancer incidence.^8^ Another study showed that deregulation of existing cell populations preceded tumour formation in an inflammatory model of bowel cancer. Chronic inflammation disrupted homeostasis in the large intestine so that the paneth cells de‐differentiated to a stem‐like population from which tumours could arise.^9^ These two studies are both examples of cancer arising from stem cells, be that an existing stem cell pool or one generated by extrinsic factors. CSC plasticity also persists during tumour growth as both stem and nonstem‐like populations are capable of inter or intraconversion in response to extrinsic signals.^10^, ^11^, ^12^ This de novo generation of the CSC phenotype has obvious implications for therapeutic strategies, however, the molecular mechanisms involved are poorly understood. Creation of CSCs by definition requires a reversible but heritable process (asymmetric division), which strongly suggests a role for epigenetic regulation and there is mounting evidence in support of this, not least the importance of epigenetics in induced pluripotency.

**FIGURE 1:**
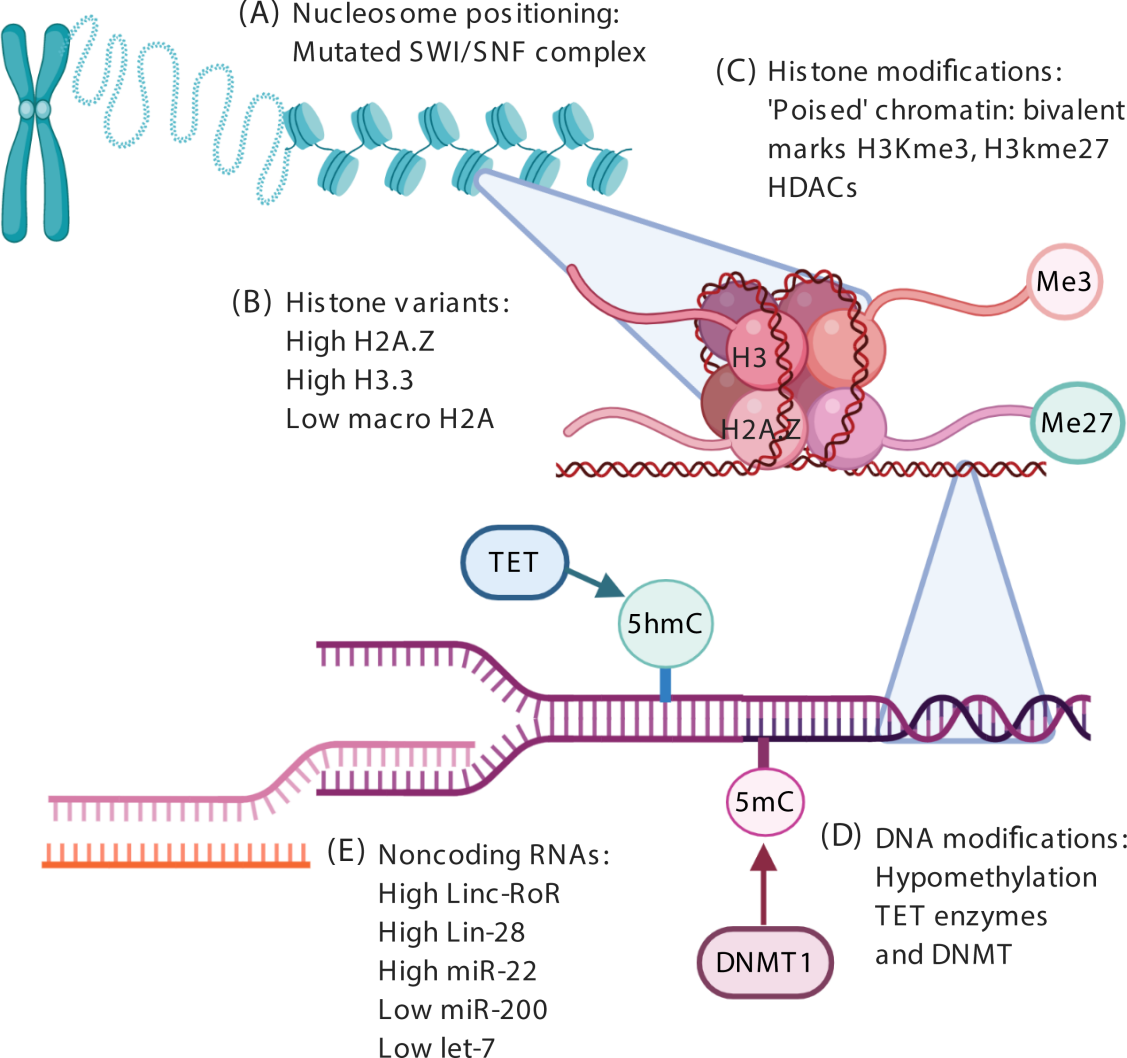
**Epigenetic regulation of cancer stem cells.** A variety of epigenetic mechanisms have been implicated in the generation of CSCs including changes in (A) nucleosome remodelling and associated complexes (eg, mutations in SWI/SNF). B, Histone variant deposition including higher H2A.Z and lower macroH2A. C, Histone modifications, in particular, bivalent histone marks D. Hypomethylation mediated by TET proteins E Altered expression of noncoding RNAs for example, high Lin28, low let‐7 [Color figure can be viewed at wileyonlinelibrary.com]

Epigenetics refers to a number of mechanisms that control the reversible regulation of gene expression by changing the chromosome without altering the DNA sequence: DNA can be altered epigenetically by methylation and demethylation of CpG nucleotides. Epigenetic changes in the overall structure of chromatin occur through at least three interrelated mechanisms: posttranslational modifications of histones, ATP‐dependent chromatin remodelling and the incorporation (or replacement) of specialised histone variants into chromatin. Finally, noncoding RNA can interact with transcriptional processes to alter gene expression. In addition to 2D processes, epigenetic regulation can also involve higher‐order chromatin organisation including promoter‐enhancer interactions, regulatory DNA loops and 3D chromatin localisation in the cell nucleus (Figure 1).

**FIGURE 2:**
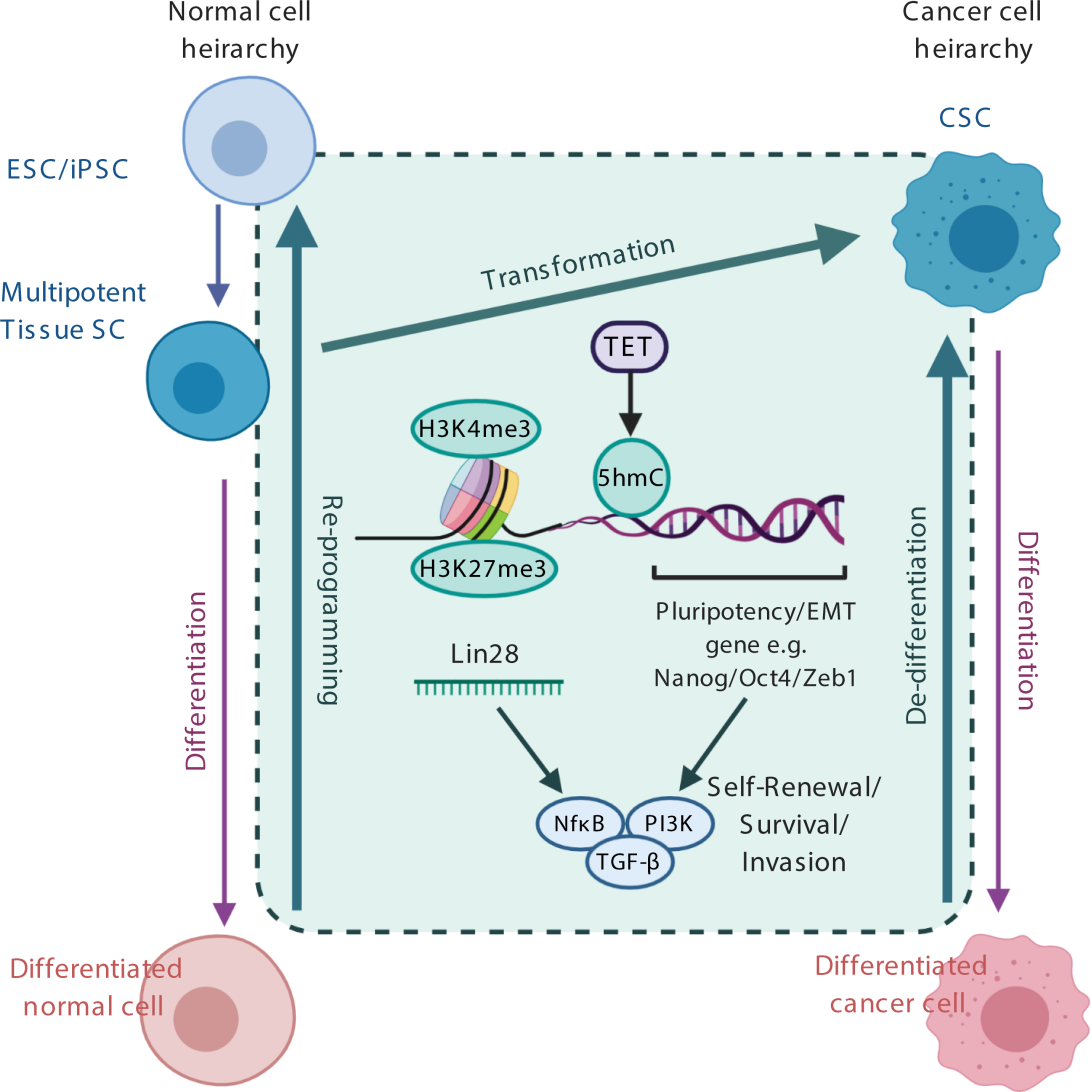
**Epigenetic parallels between reprogramming, transformation and de‐differentiation.** Transition to stem‐like states requires enhanced expression of pluripotency and EMT genes, which can be mediated at relevant loci by a combination of epigenetic processes including nucleosome loss at transcription start sites, histone variant deposition, histone modifications, in particular, bivalent histone marks to allow for rapid gene activation, demethylation by TET proteins and miRNA expression such as Lin‐28. All converge on downstream signalling pathways such as TGFβ and NfkB to promote stem‐like characteristics such as self‐renewal, survival and invasion [Color figure can be viewed at wileyonlinelibrary.com]

As these epigenetic mechanisms are important mediators of cellular identity, we will explore how the restructuring of such epigenetic barriers reinforces the stem‐like state in both normal cells and cancer, and their relevance to tumour initiation (Figure 2). Furthermore, we will discuss how the epigenetic regulation of CSCs opens up novel opportunities for cancer detection and therapeutic intervention.

## 2 DNA METHYLATION AND DEMETHYLATION IN CSC PLASTICITY

**FIGURE 3:**
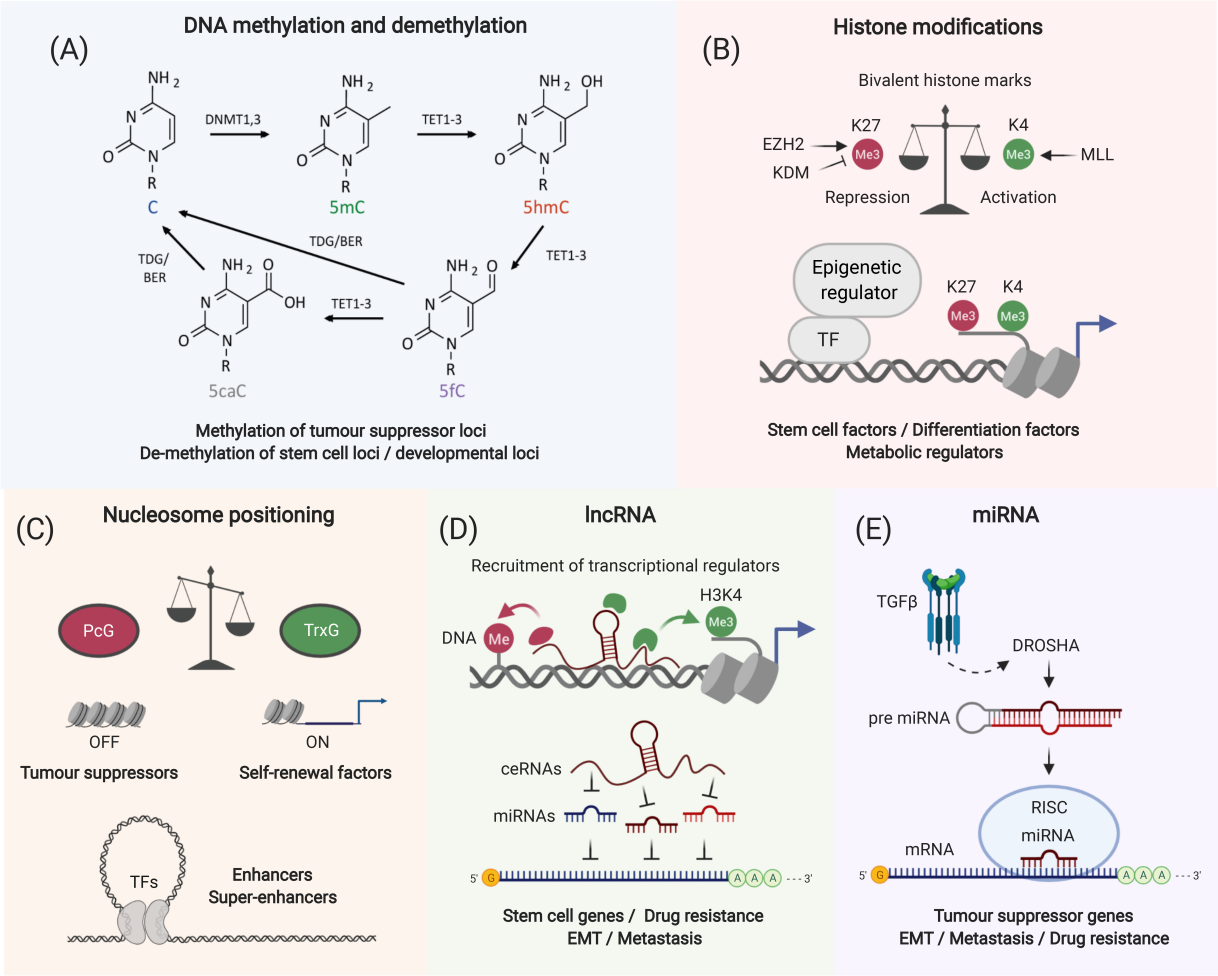
**Epigenetic mechanisms in detail.** A, Cycle of methylation and demethylation by DMNT and TET proteins, respectively. TET‐mediated demethylation generates intermediate 5hmC, 5fC and 5caC marks that are converted to unmodified cytosine by the BER pathway. B, Bivalent histone marks; the presence of both activating H3K4me3, and repressing H3K27me3 marks at certain loci allow for rapid gene activation and are characteristic of stem cells. C, Nucleosome remodelling complexes can affect gene expression at relevant loci: TrxG complexes allow expression of self‐renewal factors whereas PcG complexes prevent transcription of tumour suppressors. D, Long noncoding RNAs modify gene expression by affecting the recruitment of transcriptional regulators. E, MicroRNAs modify gene expression by directly affecting transcription [Color figure can be viewed at wileyonlinelibrary.com]

DNA methylation is a form of gene silencing that occurs mainly to CpG dinucleotides which cluster in CpG islands; areas of high CpG density usually found at promoters. To methylate cytosines, DNA methyltransferases (DNMTs) catalyse the transfer of a methyl group from cofactor S‐adenosylmethionine to the carbon of the cytosine ring to generate 5‐methylcytosine (5mC). This functions to inhibit gene expression either by recruitment of methyl‐CpG‐binding domain proteins which in turn recruit histone‐modifying and chromatin‐remodelling complexes, or by preventing the recruitment of DNA‐binding proteins, that is, transcription factors. To reactivate expression after silencing, 5‐mCs can be oxidised to 5‐hydroxymethylcytosine (5hmC) and back to the unmodified state by TET proteins and base excision repair (BER), to ultimately restore the unmethylated cytosine. TET protein‐mediated demethylation can occur via either passive or active processes. Passive demethylation results from the failure to maintain 5mC marks across cell divisions (5hmC is not a substrate for DNMTs). Active demethylation is enzymatic, whereby TET proteins further oxidise 5hmC marks to 5‐formylcytosine (5fC) and 5‐carboxylcytosine (5caC) then back to the unmodified state. 5fC bases are recognised by thymine‐DNA‐glycosylases (TDG) that excise mismatched pyrimidines that would then be replaced by an unmodified cytosine by the BER pathway. Intermediate marks (5fC or 5caC) are much less abundant than 5hmC but may also have independent functions^13^ (Figure 3A).

Methylation patterns are altered in cancer; localised hypermethylation occurs in CpG islands of the promoters of tumour suppressor genes (the so‐called “CpG island methylator phenotype”—CIMP), silencing their expression.^14^ However, global hypomethylation in intergenic regions causes oncogene activation and ultimately results in genomic instability.^15^, ^16^ What happens in the CSC subset and what methylation changes are required to generate CSCs and initiate tumour growth?

In general, CpG methylation is required for differentiation whereas demethylation is essential for induction of the pluripotent state.^17^, ^18^, ^19^ However non‐CpG methylation, although rarer, is associated with pluripotency and is lost upon development in all tissues except the brain.^20^ There is evidence to suggest that loss of methylation is also required for the generation of CSCs and tumour initiation: data produced in mouse models demonstrate that global loss‐of‐imprinting (LOI) events alone promote the onset of cancer, including loss of growth inhibition by TGFβ.^21^ CSC formation from cancer cells was found to depend on loss of methylation of the Nanog promoter via DNMT1 inhibition.^22^ Loss‐of‐function mutations in DMNT3A also led to the expansion of preleukaemic SCs, indicating a role for dysregulated DNA methylation in inducing tumour‐initiating cells.^23^

Some hypomethylation also continues to be associated with CSCs once the tumour has been established. For example, the CpG region of the CD133 gene promoter, a key gene involved in CSCs, was shown to be hypomethylated in several cancer types including breast, ovarian, colorectal and glioblastoma.^24^, ^25^, ^26^ Also, some important demethylated regions occur exclusively in CSCs compared to nonstem tumour cells. For example, in pancreatic ductal adenocarcinoma (PDAC), genes involved in CSC pathways, including GATA6, SOX9 and BMP4, were demethylated in the invasive (more stem‐like) population.^27^ Demethylation is highly important in pluripotency, both in ESCs and induced pluripotent stem cells (iPSCs). Reprogramming of somatic cells to a stem‐like state can be achieved by expression of the Yamanaka factors (Oct3/4, Sox2, Klf4 and c‐Myc), and is accompanied by epigenetic changes including demethylation. Reprogramming is dependent on TET1, which affects Nanog levels and can even act as a substitute for Oct4. However, knockout of TET1 in ESCs did not perturb the pluripotent state.^18^, ^28^, ^29^, ^30^ These studies show that TET1 is important in the establishment of pluripotency as opposed to the maintenance of this state, and suggests that TET proteins may also be involved in the generation of CSCs.

However, opposing roles for DMNT1 have been demonstrated as it can both promote or inhibit CSC formation. For example, DNMT1 was required for the initiation of colon cancer.^31^ DNMT was also important for CSC function in established leukaemia, breast, lung and PDAC tumours^32^, ^33^, ^34^, ^35^ whereas inhibition of DNMT1 promoted CSCs and EMT in prostate cancer.^36^ These discrepancies could be due to tumour‐specific effects or local hypermethylation of tumour suppressor genes which may promote the generation of CSCs in some cases.^37^

The importance of both hypomethylation and DNMT1 in CSCs suggests that specific localization of 5mC and 5hmC marks is likely to be a more important determinant of cellular identity than the overall global patterning. In particular, hypomethylation at pluripotency loci and hypermethylation at tumour suppressor genes or those involved in differentiation is important in the generation of CSCs.

## 3 HISTONE MODIFICATIONS IN CSC PLASTICITY

The amino acid residues located on the N‐ and C‐terminal tails of histones can be modified to influence gene expression, including by acetylation, methylation and ubiquitylation. When occurring on promoters or enhancer regions, these modifications confer chromatin states that affect gene expression by altering the ability of protein complexes to bind. Chromatin formation that permits protein binding and gene expression is known as euchromatin, and repressive is known as heterochromatin. These states are mediated by the Trithorax group (TrxG) proteins and Polycomb group (PcG) proteins, respectively. Modifications are named by the histone type (eg, H3) followed by the amino acid and modification, for example, K4 me3 (trimethylation on lysine 4). The most studied modifications are those that occur to histone 3 found primarily at active enhancers (H3K9ac, H3K27ac), promoters (H3K4me3) and within the bodies of actively transcribed genes (H3K36me3).^38^ Repressive marks include H3K27me3 and H3K9me3, also found on enhancers (Figure 3B).

Alterations of the histone modification landscape are widely associated with SCs with approximately one‐third of the genome differing in chromatin structure between differentiated cells and ESCs.^39^ In general, the genome of pluripotent SCs is enriched for more transcriptionally permissive euchromatin and less heterochromatin relative to somatic cells. This means that PSCs contain more acetylated chromatin and smaller regions of the repressive marks H3K9me3 and H3K27me3 relative to differentiated cells.^39^ Accordingly, certain protein modifying enzymes (eg, acetylases, demethylases) are important in PSCs and establishment of cell identity.^13^ In particular, the repressive complexes PRC1 and PRC2 are both required for induction of pluripotency.^40^

Although there are many parallels between PSCs and CSCs, histone‐modifying enzymes can have tumour‐specific effects. The subunit of the Polycomb group complex 2 (PRC2) that catalyses H3K27me3 is the histone methyltransferase EZH2 (enhancer of zeste homologue 2). EZH2 inhibition has been shown to be sufficient for tumourigenesis and is associated with the generation of CSCs in some cancers.^41^, ^42^ However, EZH2 has been found to be hyperactivated in breast cancer and PDAC.^43^, ^44^, ^45^ Loss‐of‐function mutations of the H3K27 demethylases UTX3 and JMJD3 were also found to have completely opposite effects on tumour initiation in T‐ALL whereby loss of UTX3 acted as a tumour suppressor while the loss of JMJD3 promoted tumour initiation.^46^

As well as methylation, other modifications have shown importance in CSCs. The BMI1 subunit of the polycomb repressive complex 1 (PRC1), which monoubiquitinates histone H2A on lysine 119 (H2AK119Ub1) was able to promote CSCs in leukaemia and GBM.^47^, ^48^ This was also the case for some HDACs: HDAC7 promoted tumourigenesis in the lung.^49^ In addition to histone modifiers, dysregulation of some proteins that regulate chromatin structure, such as cohesins, are involved in promotion of stem cells and the generation of CSCs in leukaemias.^50^ Furthermore, mutations that disrupt the function of chromatin‐remodelling complexes, and are found at high frequency in cancers, can cause aberrant activation of stem cell‐related pathways.^51^, ^52^

Nearly all the genes involved in ESC identity, including KLF4, Sox2, Oct4 and Nanog, are regulated by super‐enhancers; large genomic regions with very high levels of transcription factors that are highly important in controlling pluripotency and differentiation.^53^, ^54^ In turn, Sox2, Nanog and Oct4 co‐bind to various enhancers and super‐enhancers, thereby forming a self‐maintaining gene circuitry that maintains pluripotency. Aberrant activity of histone modifiers can impair the function of enhancer regions and effect cell identity, with certain modifications promoting the acquisition of stem‐like cells.^55^, ^56^ One study highlights the importance of enhancer reprogramming in the generation of stem cell identity by an oncogenic event. Overexpression of the oncogenic MYC in luminal human mammary epithelial cells (HMECs) led to downregulation of transcription factors and consequent loss of activity at enhancer regions important for maintaining luminal identity. At the same time, new enhancer regions were activated inducing transcriptional reprogramming to the stem‐like state. This enabled the HMECs to acquire stem‐like features including self‐renewal and multipotency.^57^

Chromatin at promoters and enhancers can be classed as active, repressed or poised. Poised regions are bivalent in terms of histone modifications as they contain both H3K27me (repressive) and H3K4me (activating) histone marks. In ESCs, the majority of bivalency occurs in the promoters of transcription factors and half of bivalent domains have binding sites for pluripotency transcription factors.^58^ In ESCs, bivalency tends to be lost upon differentiation with some enhancers becoming active (loss of H3K27me3, gain of H3K27ac) and some become repressed (H3K27me3 enrichment), this occurring in a cell type‐specific manner.^59^ As the fluid interconversion between cellular states (normal and stem) depends on the capacity of a cell to switch on and off cell‐specific transcriptional programs, it is not surprising that poised chromatin is also important for determining cell identity and plasticity in CSCs. Indeed CSCs possess a more plastic and dynamic chromatin formation than differentiated cells.^60^ In AML CSCs, genes involved in stem cell identity were bivalently marked with both H3K4me3 and H3K27me3, with H3K4me3 marks lost during differentiation.^61^ Bivalent marks at pluripotency loci have also been identified in solid cancers.^62^, ^63^ In theory, poised chromatin can allow cellular identity to switch in any direction, that is, not only differentiation but also de‐differentiation, and may therefore be important in the generation of CSCs. Bivalency also allows for the highly dynamic state so characteristic of pluripotency, and may be the single most important epigenetic process for conferring CSC plasticity. A direct link between CSC generation and bivalency was demonstrated in breast cancer where CSCs marked by high CD44 expression were generated from CD44‐low cells by TGFβ stimulation. This process was dependent on poised, bivalent chromatin at the promoter of the ZEB1 gene, and conversion of bivalent to repressive marks rendered CD44‐low cells insensitive to TGFβ.^64^

## 4 NUCLEOSOME POSITIONING AND HISTONE VARIANTS IN CSC PLASTICITY

The basic unit of chromatin is the nucleosome, which is formed of 147 bp of DNA wrapped around eight histones, two each of histones H2A, H2B, H3 and H4. These appear like beads on a string of chromatin. Gene expression is regulated at the level of chromatin structure in an ATP‐dependant process by chromatin modifiers which act to remove or slide assembled nucleosomes along with the DNA, and can also exchange histone H2A‐H2B dimers with dimers of histone variants. The presence of nucleosomes naturally represses gene expression by preventing the access of transcription factors. The absence or loss of nucleosomes at a transcription start site (nucleosome‐free region [NFR]) allows for assembly of the transcription machinery and rapid activation of gene expression.^65^, ^66^ The position of nucleosomes must therefore be precisely regulated at promoters, enhancers and repressors. This is achieved by four known families of ATP‐dependent chromatin remodelling complexes; switch/sucrose nonfermenting (SWI/ SNF), imitation switch (ISWI), inositol requiring 80 (INO80) and those with a NuRD/Mi‐2/CHD helicase binding domain.^52^, ^67^, ^68^, ^69^ Chromatin remodelling complexes use ATP hydrolysis to catalyse the assembly, sliding and ejecting of nucleosomes.^65^ DNA‐sequence specificity is achieved by interaction with transcription factors. How nucleosome reorganisation is achieved is not fully understood but it is thought that remodelling complexes cause DNA to loop or twist to disrupt connection with histones and thus forcing translocation across a nucleosome. Chromatin modifiers can also interact with methylated DNA and covalent histone modifications to affect global gene expression patterns and chromatin architecture^70^, ^71^ (Figure 3C).

The chromatin architecture of SCs differs greatly from that of differentiated cells. While small‐scale chromatin changes at DNA regulatory elements occur during transcriptional regulation in most cells, extensive remodelling of chromatin structure is required for cellular differentiation during embryonic development.^72^, ^73^, ^74^ In particular, the SWI/SNF remodelling complex is known to be important in ESCs and development: SWI/SNF complexes containing the ARID1A protein can remodel chromatin to inhibit expression of the pluripotency genes *Sox2* and *Oct4*, and promote differentiation to mesoderm, but not ectoderm.^75^ Nucleosome positioning is also important in the reprogramming of somatic cells to pluripotency. Nucleosomal landscapes of induced PSCs become extensively altered during reprogramming, in particular, nucleosome occupancy is reduced at enhancers which colocalise with binding sites of key pluripotency transcription factors, including Klf4, Oct4, Sox2 and c‐Myc.^76^

As nucleosome repositioning is required for the generation of the pluripotent state it may be important for the formation of CSCs. This may occur via aberrant functions of ATP‐dependent chromatin remodelers, which has also been linked to cancer, especially in terms of the SWI/SNF complex, which is frequently found mutated.^77^, ^78^, ^79^ In leukaemia, the SWI/SNF complex appears to function as it does in PSCs where the ATPase subunit BRG1 is required for nucleosome repositioning at the Myc enhancer to promote Myc expression, leading to enhanced self‐renewal.^80^, ^81^ However, in lung cancer, loss of either BRG1 or BRM ATPase subunits can promote cancer development.^82^ The SNF5 (BAF47) subunit of the SWI/SNF complex is considered a tumour suppressor and its inhibition is sufficient to drive malignant transformation by inactivating p16 and p21. In the case of the rare paediatric rhabdoid tumour, SNF5 mutation is an inherited lesion that is considered the sole cause of this highly aggressive cancer.^83^ Rhabdoid tumours are poorly differentiated and highly metastatic suggesting a high proportion of CSC features. This strongly links aberrant SWI/SNF‐dependent nucleosome remodelling with CSC generation. This process may also play a role in CSC generation in other cancers as mutations of *SNF5* have been identified in epithelioid sarcoma and renal medullary carcinoma.^84^, ^85^

ATP‐dependent chromatin remodelling complexes can also regulate transcription via incorporation of histone variants into nucleosomes.^86^ There are eight histone variants of H2A, and six of H3, which are deposited in specific locations along the genome. Histone variants can influence gene expression by directly altering the structure and stability of nucleosomes, or by recruiting readers of histone modifications to induce local chromatin changes. For example, nucleosomes that contain the histone variant H2A.Bbd, bind less DNA and are not as stable, resulting in less compact chromatin. Less stable nucleosomes including those that contain H2A.Bbd, H2A.Z or H3.3 are localised at active promoters, enhancers and insulators, and may serve to prevent the formation of stable nucleosomes around these regulatory regions and facilitate transcription.^87^ In contrast, nucleosomes containing the histone variant macroH2A are relatively more stable and inhibit transcription.^88^

High mobility and exchange of histone variants is a key feature of SCs and this dynamism is thought to contribute to SC plasticity.^89^, ^90^ The expression of histone variants is tightly regulated in ES cells and during development, and variants have specialised roles in cell fate decisions and lineage‐specification.^73^, ^91^ These variants, in turn, have a corresponding influence on reprogramming: those expressed in early development (the less stable H3.3, TH2A and TH2B) facilitate transcription and promote reprogramming whereas those expressed in somatic cells (the more stable macroH2A) inhibit transcription and prevent the induction of pluripotency.^91^ As the histone variant macroH2A is considered an epigenetic barrier to reprogramming, a tumour suppressive function would be expected in cancer.^92^ Accordingly, overexpression of macroH2A reduced metastatic potential of melanoma and inhibition of macroH2A1 generated CSCs in hepatocellular carcinoma.^93^, ^94^ Importantly, inhibition of macroH2A1 in bladder cancer increased stem‐like cells.^95^ In contrast, the less stable H2A.Z and H3.3 variants promote transcription and reprogramming, and both have been found to be overexpressed in cancer.^96^ H2A.Z correlates with poor prognosis in melanoma, breast cancer and hepatocellular carcinoma where it has been linked with EMT.^97^, ^98^, ^99^ However, the role of H3.3 appears to differ in adult GBM where it is repressed in SCs and its overexpression impairs self‐renewal. Furthermore, repression of H3.3 in nonstem cancer cells is sufficient to induce cell plasticity and generate a CSC state.^100^

These findings suggest that the overexpression of labile histone variants such H2A.Z (and in some cases H3.3), coupled with the reduction of stable histone variants such as macroH2A could contribute to cancer cell plasticity and the generation of CSCs.

### 4.1 Noncoding RNA and CSC plasticity

Noncoding RNAs (ncRNA) are transcribed from regions of the genome that do not encode for proteins. The resulting RNA transcripts function to regulate the expression of protein‐coding genes and are therefore essential for control of cellular function and identity. ncRNAs can be divided into two major groups based on their size: small ncRNAs being 200 nucleotides or less, and long ncRNAs (lncRNAs) 200 nucleotides or more. Small ncRNAs can be further subcategorized based on length, function and subcellular localization and include microRNAs (miRNAs) and short interfering RNAs (siRNAs) amongst others.^101^ Long and short ncRNAs regulate gene expression by different modes of action.

### 4.2 Long ncRNAs

LncRNAs can physically associate with DNA or proteins to either promote or repress gene expression. To promote transcription, lncRNAs can function as guides or scaffolds for the assembly of protein complexes at specific loci. To repress transcription lncRNAs can function as decoys, binding and preventing functions of RNA or protein targets^102^ (Figure 3D). Many lncRNAs are known to be involved in the regulation of pluripotency and cell fate transitions and are important in many types of adult stem cells. In particular, long intergenic ncRNAs (lincRNAs) which reside in gene deserts have tissue‐specific expression patterns that make them key players in the establishment of cellular identity. Knockdown of those lincRNAs associated with pluripotency results in differentiation of PSCs.^103^ Furthermore, the expression of many lncRNAs is altered during the early stages of reprogramming to iPSCs.^104^ Some lncRNAs associated with pluripotency have also been found to be upregulated in CSCs, for example, H19, which is involved in both mesenchymal SCs and glioblastoma CSCs.^105^, ^106^ Importantly, many lncRNAs have been implicated in the generation of CSCs via roles in cellular transformation and EMT. In particular, the lncRNA LINK‐RoR (regulator of reprogramming) is elevated in iPSCs and modulates iPS‐mediated reprogramming.^107^ LINK‐RoR is also more highly expressed in CSCs as compared to more differentiated cancer cells and is important in a wide range of cancer types.^108^, ^109^, ^110^, ^111^, ^112^, ^113^ Furthermore, LINK‐RoR also induces EMT and may therefore be involved in the conversion of cancer cells to CSCs during tumourigenesis.^109^, ^114^ Many other lncRNAs play an important role in EMT. In particular, the lncRNA HOTAIR is involved in the recruitment of PRC2 to Hox genes during development and is also upregulated in TGF‐beta1‐responsive cancer cells during EMT.^115^, ^116^

### 4.3 Micro RNAs

MiRNAs function by recognising small sequences (6‐8 nucleotides) at the 3′‐untranslated region of multiple target mRNAs.^117^ To do this, miRNAs are assembled in the RNA‐induced silencing complex (RISC), where complementarity between the miRNA and mRNA target sequence results in mRNA cleavage. If partial complementarity occurs, deadenylase complexes are recruited which remove or shorten the mRNA poly‐A tail to impair translation^101^ (Figure 3E).

The importance of miRNAs in embryonic stem cells (ESCs) has been established by deletion of Dicer1 and DGCR8 (both critical for miRNA biogenesis) in mouse models. Dicer1 deletion is embryonic lethal whereas DGCR8‐deficient mice have impaired differentiation due to failure to silence the stem markers, Oct4, Sox2 and Nanog.^118^, ^119^ This phenotype can be rescued by expression of the let‐7 family miRNAs, which are not expressed in ESCs and appear during differentiation.^120^, ^121^ Let7 miRNA expression is controlled by a negative feedback loop with Lin‐28, which is a marker of pluripotency and is also important in reprogramming.^122^ In PSCs and cancer cells, Lin‐28 binds to let‐7 precursors to block their maturation, whereas the let‐7 family miRNAs target Lin‐28 for degradation in differentiated cells.^121^

Let7 is also known as a negative regulator of CSCs especially in breast cancer where, as expected, let‐7 was linked with differentiation. Inhibition of let‐7 promoted CSC properties, mediated by increased RAS and HMGA2 expression.^123^ In turn, Lin‐28 has been shown to promote CSCs in breast and prostate cancer,^124^, ^125^, ^126^, ^127^ and even induce transformation in multiple cancers.^128^, ^129^, ^130^ It was found that transformation of a normal breast cell line by the Src oncoprotein required an epigenetic switch whereby the resulting upregulation of NFkB by src directly activated transcription of Lin‐28, which in turn reduced let‐7 expression. Low let‐7 expression allowed for elevated levels of IL6, which in turn reactivated NFkB. This epigenetic positive feedback loop was found to be essential for maintaining the transformed state of breast cancer cells.^128^ These findings strongly suggest that the Lin‐28/ let‐7 miRNA axis may control the generation of CSCs in cancer as it does with pluripotency of noncancerous cells.

As well as directly affecting the transcription of pluripotency‐associated genes, miRNAs can also affect CSC biology via cross‐talk with other CSC pathways and epigenetic mechanisms. For example, the maturation of miR‐21 via a DROSHA and RNA helicase p68 complex is induced by TGF‐β signalling.^131^ The up‐regulation of miR‐21 promotes cancer invasion and metastasis by negatively regulating the expression of the tumour suppressor gene PDCD4.^132^ Furthermore, miR‐22 promotes SCs in breast cancer through direct inhibition of TET activity, thus preventing demethylation of the mir‐200 promoter and silencing mir‐200 expression.^133^ miRNA expression in SCs is also regulated by bivalent promoters. In particular, lineage‐specific miRNAs appear to be controlled in this manner, suggesting that an important mechanism underlying cellular plasticity is mediated by miRNAs.^134^

## 5 THE ROLE OF EMT‐MET PLASTICITY IN THE EPIGENETIC REGULATION OF CSCS

Many studies have associated EMT with the generation of CSCs.^135^, ^136^ However two distinct but interconverting populations of CSCs have been identified in some cancers including breast and pancreatic; one being more epithelial‐like and one more mesenchymal‐like.^6^, ^12^ The functional relevance of two CSC populations is thought to lie in the fact that metastasis requires phenotypic plasticity: mesenchymal‐like properties are required for cellular dissemination whereas epithelial properties are required for attachment and proliferation within the foreign niche.^137^ EMT‐MET plasticity can be conferred and maintained by epigenetic mechanisms. For example, a TGF‐β‐induced EMT resulted in a downregulation of p53 and the miRNAs 200c and 183, and concomitant elevation of the stem cell‐related genes Bmi1 and Klf4, which could be rescued by overexpression of miR‐ 200c or p53.^138^ Bivalent histone markings could also mediate EMT‐MET plasticity as poised chromatin allows for flexible determination of gene expression.

CSC generation by EMT may also be influenced by the localised tissue environment via epigenetic modification. Cells present in the tumour CSC niche such as stromal and immune cells produce cytokines and growth factors which promote CSC characteristics. For example, tumour‐associated macrophages (TAMs) secrete VEGF and IL6, and T cells secrete IL17 and TGF‐β, all of which are able to promote CSCs via EMT.^139^, ^140^ In addition, myeloid‐derived stem cells in the TME secrete IL6 and nitric oxide that promote CSC characteristics via a Stat‐3 and mir101‐mediated EMT.^141^, ^142^ Environmental cross‐talk occurs both ways; epigenetic mechanisms in CSCs also alter their interaction with cells in the TME. For example, hypermethylation at the Tap promoter enhances the ability of CSCs to evade destruction by immune cells.^143^

## 6 TRANSLATIONAL RELEVANCE OF CSC EPIGENETICS

**FIGURE 4:**
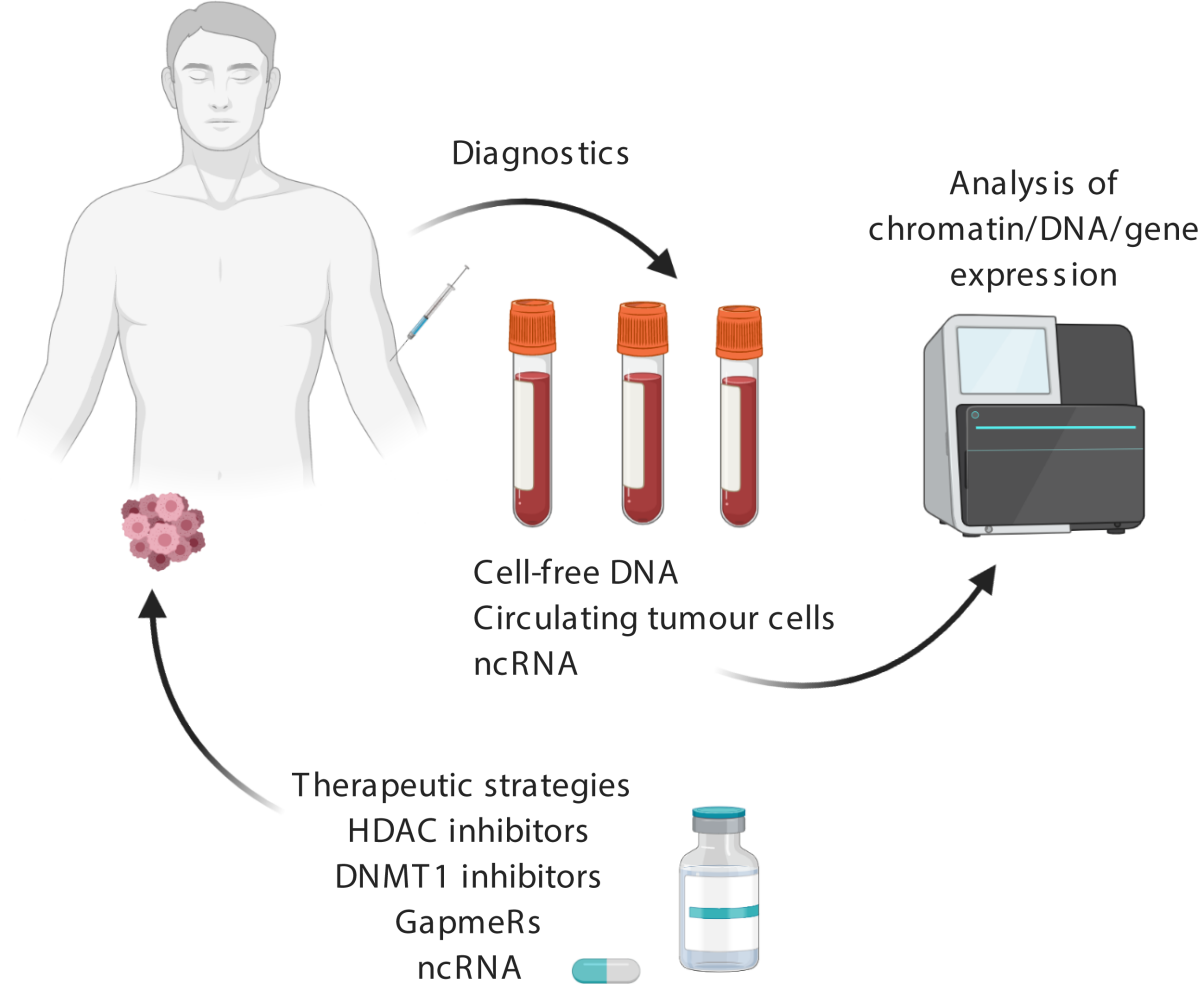
**Translational relevance of epigenetic processes.** Epigenetic mechanisms involved in CSC generation reveal factors with potential both as biomarkers for early diagnosis and as therapeutic targets. Early diagnosis could be achieved by analysis of cell‐free DNA, circulating tumour cells or noncoding RNAs for the presence of characteristic epigenetic marks. Epigenetic marks could also be used as targets for therapeutic agents, such as DMNT1 or HDAC inhibitors. Alternatively, ncRNAs or gapmeRs could be used as therapeutics to modify gene expression [Color figure can be viewed at wileyonlinelibrary.com]

Many epigenetic aberrations identified as being involved in the generation of CSCs are likely early markers of cancer and therefore potentially useful as early detection methods. Early cancer detection could be achieved by analysis of patient blood samples, which is considered a relatively noninvasive procedure compared to a biopsy. Patient blood can contain cancer cells and cell‐free DNA, which could be identified by characteristic genetic and epigenetic signatures as biomarkers of the cancerous state. Should such a process be found to be accurate, it may facilitate the detection of tumour signatures even when the tumour is not large enough to be picked up on a scan. This technique was originally designed for the identification of large genomic alterations, however, advances in the understanding of epigenetic aberrations in cancer have led to the pursuit of an epigenetic signature that may define the cancerous state. Although these may differ between cancers, epigenetic signatures associated with the generation of CSCs are likely candidates across cancer types due to the requirement for CSC generation in tumour initiation (Figure 4).

Most approaches have focussed on the methylation status of particular gene promoters but it is likely that the methylation status of several promoters would be required to serve as an accurate and reliable signature. For example, hypermethylation of the combination of four genes: *BCAT1*, *CDO1*, *TRIM58* and *ZNF177*, has been shown to predict lung cancer.^144^ Recently, methylation patterns were used to detect and localise over 50 different cancer types.^22^ Hypomethylation is a characteristic signature of cancer and CSCs, and considered an early event in tumourigenesis. Therefore, a global hypomethylation profile, defined by the presence of 5‐hmC marks, may be a useful sign of tumour presence. In particular, hypomethylation of SC‐associated genes such as Oct4, Sox2 and Nanog, together with or methylation of certain differentiation markers or tumour suppressor genes such as p53 may indicate the presence of CSCs and therefore early cancerous lesions.

An increase in histones and their associated covalent modifications have also been identified in the blood of cancer patients.^145^ Bivalent histone signatures may be a promising avenue of exploration due to their specific association with CSCs. Unexpectedly, nucleosome positioning inferred from whole‐genome sequencing of plasma DNA correlates with RNA levels and furthermore with RNA in tumour tissue. This suggests that nucleosome positioning could also accurately reflect gene expression and be used to predict tumour incidence.^146^

A useful form of epigenetic cancer detection is ncRNA. Noncoding RNAs are readily secreted by the tumour into the microenvironment and subsequently reach the bloodstream and even distant tissue sites. LncRNAs and miRNAs have been detected in blood, urine and saliva, all of which facilitate diagnostic testing and many have been identified as potential biomarkers.^147^, ^148^ Biomarkers have been identified through comparison of the pre and postoperative state, that is, a potentially accurate cancer biomarker should decrease upon removal of the tumour. Such candidates include mir‐221 and mir‐375 in breast and pancreatic cancer^149^, ^150^ and mir‐20, mir‐21, mir‐145 and mir‐223 in early‐stage lung cancer.^151^ Some miRNAs may even be able to distinguish certain subtypes of cancer, for example, lower levels of miR‐16‐5p, miR‐21‐5p and miR‐199a‐5p were found to be associated specifically with the presence of triple‐negative breast cancer.^152^ Many of those miRNAs found upregulated in cancer are also associated with CSCs. For example, mir‐221 was found to promote the generation of CSCs in breast cancer. Interestingly, this occurred via its role in the downregulation of DMNT3b, hence providing a link between a miRNA and reduced methylation, also associated with CSCs.^153^ Mi‐21 is also a promoter of CSCs in many cancers especially colorectal.^154^, ^155^ These associations suggest that a role in CSCs may define what makes a useful and specific epigenetic biomarker for cancer.

Epigenetic targeting is an exciting area of translational cancer research offering many novel opportunities for therapeutic intervention. Many studies have explored the use of existing and novel compounds to target epigenetic marks to impair cancer cell growth and elicit an improved clinical response (Figure 4).

HDAC inhibitors are already widely considered to have anticancer effects and some are in clinical use. For example, SAHA (vorinostat) is approved for T‐cell lymphoma and many others are in clinical trials for various malignancies either alone or in combination with other therapeutics.^156^ Despite promising anticancer effects, results have been mixed and in some cases accompanied by substantial toxicity.^157^, ^158^ However, preclinical studies have shown that a range of HDAC inhibitors can preferentially target CSCs in cancer cell lines. In breast cancer, these effects were attributed specifically to inhibition of the HDACs 1 and 7.^159^ However, the HDAC inhibitor valproic acid, was actually able to increase the number of CSCs in breast cancer via inducing de‐differentiation to the stem‐like state (ie, CSC generation).^160^ These effects were mediated by the promotion of wnt‐signalling. These conflicting results may reflect the specificities of different HDAC inhibitors, or the nonspecific effects of HDAC inhibitors in general, and may explain conflicting clinical data and toxicities. While there may be potential in pursuing HDAC inhibition as a CSC‐targeting approach, much further work is required to elucidate underlying mechanisms of action and avoid adverse effects. An alternative approach to targeting histone acetylation has been sought through bromodomain inhibitors. Bromodomains (BRDs) are readers of histone acetylation that target chromatin‐modifying enzymes to specific genomic sites to regulate transcription. The bromodomain and extra terminal (BET) family is made up of four different proteins that are important in cancer and also function as cell cycle regulators. The first developed inhibitor of the BET bromodomain family was JQ1.^161^, ^162^ A combination of JQ1 and the HDAC inhibitor SAHA promoted apoptosis in a number of cancers including PDAC. JQ1 also promoted apoptosis and cell‐cycle arrest of CSCs in glioma.^163^, ^164^

Targeting of histone modifications may also be achieved through LSD1 inhibitors. Lysine‐specific demethylase 1A (LSD1) removes methyl groups from the histone 3 proteins (H3K4me2/1 and H3K9me2/1) resulting in transcriptional repression or activation, respectively.^165^ LSD1 is overexpressed in cancer, where it functions to inhibit differentiation and enhance proliferation, invasiveness and cell motility.^166^ Effective LSD1 inhibitors have been developed by mimicking the lysine groups of the LSD phenyl ring at two positions (NCL‐1) or (NCL‐2).^167^ NCL‐1 analogues have exhibited good anticancer activities across solid tumours including against CSCs.^168^

Histone modification could also be targeted by inhibition of PRC components BMI‐1 or EZH2. BMI‐1 is a key regulatory component of (PRC1) and its function is strongly associated with the maintenance of CSCs.^169^, ^170^ Two small molecule inhibitors of BMI‐1, PTC‐209 and QW24, both impaired the self‐renewal of colorectal CSCs.^171^ EZH2 is an enzymatic catalytic subunit of polycomb repressive complex 2 (PRC2), which is also associated with CSC function.^172^ Several inhibitors of EZH2 methyltransferase activity have been developed with a 2‐pyridone core designed to occupy the binding pocket for cosubstrate S‐adenosyl‐methionine (SAM) in EZH2. GSK343 is one such SAM‐competitive inhibitor, which has been shown to inhibit stemness in patient‐derived glioma lines.^173^ However, targeting of EZH2 may be unpredictable as it has been shown to have opposing effects across cancer types.^41^, ^42^, ^43^, ^44^, ^45^, ^46^

Although an increasing number of ncRNAs are known to be involved in CSCs and cancer in general, and are therefore attractive drug targets, it is considered difficult to specifically target aberrant mRNAs or RNA‐protein interactions with small molecules. Nevertheless, due to the high promise of such targets, a number of studies have attempted therapeutic modulation. The miRNA lin28 which is very important in CSC generation has been found to be targeted by the compound “N‐methyl‐N‐[3‐(3‐methyl[1,2,4]triazolo[4,3‐b]pyridazin‐6‐yl)phenyl]acetamide”, which acts by blocking the interaction between lin‐28 and let‐7. This compound impaired Lin‐28, rescued let‐7 function and resulted in differentiation in ESCs, and reduced tumorsphere formation in cancer cell lines. It is likely that the reduction in tumoursphere formation was caused by differentiation of CSCs.^174^ An alternative approach is the administration of synthetic miRNAs to restore functions lost in cancer, for example, the introduction of tumour suppressor miRNAs.^175^

Another way of targeting DNA and RNA moieties is by using peptide nucleic acids (PNA). These are synthetic analogues of DNA that are highly stable and have a hybridization affinity toward natural DNA and RNA, and are therefore a potential approach for the modification of gene expression and epigenetic states.^176^ This approach has been applied to the targeting of the lncRNA HOTAIR to block its interaction with EZH2, resulting in reduced invasiveness and increased sensitivity to chemotherapy.^177^ These effects strongly suggest inhibition of the stem‐cell compartment.

Despite promising results, therapeutic targeting of epigenetic marks should proceed with caution. A therapeutic strategy designed to target epigenetic readers and writers is by definition not a specific approach. Such an intervention would elicit gene expression changes on a global scale and is likely to incur off‐target and adverse effects. Therefore much further investigation is required to identify a clinically viable approach. Epigenetic targeting that focuses on a specific gene or pathway changes (eg, miRNA and lncRNA) may be more easily targeted to CSC inhibition and therefore have much greater potential for efficacy.

## 7 CONCLUSIONS AND FUTURE DIRECTIONS

The epigenetic processes involved in CSC generation have substantial implications for tumour initiation, metastasis and relapse. Many studies have focussed on the elucidation of mechanistic links between epigenetic processes and CSCs, and have identified attractive targets for cancer prevention, diagnosis and therapy. However, as always, these studies have highlighted many areas in which further investigation is required. In particular:

- To obtain a greater understanding of the epigenetic mechanisms which drive CSC formation from normal cells.
- To determine whether the mechanisms of CSC generation differ when deriving from normal or cancerous cells and their associated stem and nonstem subpopulations. Epigenetic mechanisms involved in the emergence of CSCs will likely differ depending on from which population they are derived, that is, from a normal cell, normal stem cell, pre‐CSC or bulk tumour cell, as each of these identities has distinct epigenetic profiles.
- To ascertain the relative contribution of genetic and epigenetic effects to tumour initiation; can epigenetic events alone initiate the transformed state?
- To determine which are the most important environmental signals involved in CSC generation and how these can be modified or targeted for prevention and treatment.

## CONFLICT OF INTEREST

The authors note no conflict of interest.

## References

[1] Bonnet D , Dick JE . Human acute myeloid leukemia is organized as a hierarchy that originates from a primitive hematopoietic cell. Nat Med. 1997;3:730‐737. 9212098 10.1038/nm0797-730

[2] Al‐Hajj M , Wicha MS , Benito‐Hernandez A , Morrison SJ , Clarke MF . Prospective identification of tumorigenic breast cancer cells. Proc Natl Acad Sci USA. 2003;100:3983‐3988. 12629218 10.1073/pnas.0530291100 PMC153034

[3] Singh SK , Hawkins C , Clarke ID , et al. Identification of human brain tumour initiating cells. Nature. 2004;432:396‐401. 15549107 10.1038/nature03128

[4] O'Brien CA , Pollett A , Gallinger S , Dick JE . A human colon cancer cell capable of initiating tumour growth in immunodeficient mice. Nature. 2007;445:106‐110. 17122772 10.1038/nature05372

[5] Ricci‐Vitiani L , Lombardi DG , Pilozzi E , et al. Identification and expansion of human colon‐cancer‐initiating cells. Nature. 2007;445:111‐115. 17122771 10.1038/nature05384

[6] Hermann PC , Huber SL , Herrler T , et al. Distinct populations of cancer stem cells determine tumor growth and metastatic activity in human pancreatic cancer. Cell Stem Cell. 2007;1:313‐323. 18371365 10.1016/j.stem.2007.06.002

[7] Reya T , Morrison SJ , Clarke MF , Weissman IL . Stem cells, cancer, and cancer stem cells. Nature. 2001;414:105‐111. 11689955 10.1038/35102167

[8] Zhu L , Finkelstein D , Gao C , et al. Multi‐organ mapping of Cancer risk. Cell. 2016;166:1132‐46.e7. 27565343 10.1016/j.cell.2016.07.045 PMC5067024

[9] Schmitt M , Schewe M , Sacchetti A , et al. Paneth cells respond to inflammation and contribute to tissue regeneration by acquiring stem‐like features through SCF/c‐kit signaling. Cell Rep. 2018;24:2312‐28.e7. 30157426 10.1016/j.celrep.2018.07.085

[10] Chaffer CL , Brueckmann I , Scheel C , et al. Normal and neoplastic nonstem cells can spontaneously convert to a stem‐like state. Proc Natl Acad Sci USA. 2011;108:7950‐7955. 21498687 10.1073/pnas.1102454108 PMC3093533

[11] Gupta PB , Fillmore CM , Jiang G , et al. Stochastic state transitions give rise to phenotypic equilibrium in populations of cancer cells. Cell. 2011;146:633‐644. 21854987 10.1016/j.cell.2011.07.026

[12] Liu S , Cong Y , Wang D , et al. Breast cancer stem cells transition between epithelial and mesenchymal states reflective of their normal counterparts. Stem Cell Rep. 2014;2:78‐91. 10.1016/j.stemcr.2013.11.009 PMC3916760 24511467

[13] Boland MJ , Nazor KL , Loring JF . Epigenetic regulation of pluripotency and differentiation. Circ Res. 2014;115:311‐324. 24989490 10.1161/CIRCRESAHA.115.301517 PMC4229506

[14] Baylin S , Bestor TH . Altered methylation patterns in cancer cell genomes: cause or consequence? Cancer Cell. 2002;1:299‐305. 12086841 10.1016/s1535-6108(02)00061-2

[15] Gaudet F , Hodgson JG , Eden A , et al. Induction of tumors in mice by genomic hypomethylation. Science. 2003;300:489‐492. 12702876 10.1126/science.1083558

[16] Baylin SB , Jones PA . Epigenetic determinants of Cancer. Cold Spring Harb Perspect Biol. 2016;8(9):a019505. 10.1101/cshperspect.a019505 PMC5008069 27194046

[17] Leitch HG , McEwen KR , Turp A , et al. Naive pluripotency is associated with global DNA hypomethylation. Nat Struct Mol Biol. 2013;20:311‐316. 23416945 10.1038/nsmb.2510 PMC3591483

[18] Jackson SA , Sridharan R . The nexus of Tet1 and the pluripotency network. Cell Stem Cell. 2013;12:387‐388. 23561438 10.1016/j.stem.2013.03.007

[19] Suelves M , Carrio E , Nunez‐Alvarez Y , Peinado MA . DNA methylation dynamics in cellular commitment and differentiation. Brief Funct Genomics. 2016;15:443‐453. 27416614 10.1093/bfgp/elw017

[20] Lister R , Mukamel EA , Nery JR , et al. Global epigenomic reconfiguration during mammalian brain development. Science. 2013;341:1237905. 23828890 10.1126/science.1237905 PMC3785061

[21] Holm TM , Jackson‐Grusby L , Brambrink T , Yamada Y , Rideout WM 3rd , Jaenisch R . Global loss of imprinting leads to widespread tumorigenesis in adult mice. Cancer Cell. 2005;8:275‐285. 16226703 10.1016/j.ccr.2005.09.007

[22] Liu S , Cheng K , Zhang H , et al. Methylation status of the Nanog promoter determines the switch between Cancer cells and Cancer stem cells. Adv Sci. 2020;7:1903035. 10.1002/advs.201903035 PMC7055559 32154082

[23] Mayle A , Yang L , Rodriguez B , et al. Dnmt3a loss predisposes murine hematopoietic stem cells to malignant transformation. Blood. 2015;125:629‐638. 25416277 10.1182/blood-2014-08-594648 PMC4304108

[24] Yi JM , Tsai HC , Glockner SC , et al. Abnormal DNA methylation of CD133 in colorectal and glioblastoma tumors. Cancer Res. 2008;68:8094‐8103. 18829568 10.1158/0008-5472.CAN-07-6208 PMC2744404

[25] Baba T , Convery PA , Matsumura N , et al. Epigenetic regulation of CD133 and tumorigenicity of CD133+ ovarian cancer cells. Oncogene. 2009;28:209‐218. 18836486 10.1038/onc.2008.374

[26] Kagara N , Huynh KT , Kuo C , et al. Epigenetic regulation of cancer stem cell genes in triple‐negative breast cancer. Am J Pathol. 2012;181:257‐267. 22626806 10.1016/j.ajpath.2012.03.019

[27] Sun L , Mathews LA , Cabarcas SM , et al. Epigenetic regulation of SOX9 by the NF‐kappaB signaling pathway in pancreatic cancer stem cells. Stem Cells. 2013;31:1454‐1466. 23592398 10.1002/stem.1394 PMC3775871

[28] Costa Y , Ding J , Theunissen TW , et al. NANOG‐dependent function of TET1 and TET2 in establishment of pluripotency. Nature. 2013;495:370‐374. 23395962 10.1038/nature11925 PMC3606645

[29] Gao Y , Chen J , Li K , et al. Replacement of Oct4 by Tet1 during iPSC induction reveals an important role of DNA methylation and hydroxymethylation in reprogramming. Cell Stem Cell. 2013;12:453‐469. 23499384 10.1016/j.stem.2013.02.005

[30] Doege CA , Inoue K , Yamashita T , et al. Early‐stage epigenetic modification during somatic cell reprogramming by Parp1 and Tet2. Nature. 2012;488:652‐655. 22902501 10.1038/nature11333 PMC5176099

[31] Morita R , Hirohashi Y , Suzuki H , et al. DNA methyltransferase 1 is essential for initiation of the colon cancers. Exp Mol Pathol. 2013;94:322‐329. 23064049 10.1016/j.yexmp.2012.10.004

[32] Trowbridge JJ , Sinha AU , Zhu N , Li M , Armstrong SA , Orkin SH . Haploinsufficiency of Dnmt1 impairs leukemia stem cell function through derepression of bivalent chromatin domains. Genes Dev. 2012;26:344‐349. 22345515 10.1101/gad.184341.111 PMC3289882

[33] Pathania R , Ramachandran S , Elangovan S , et al. DNMT1 is essential for mammary and cancer stem cell maintenance and tumorigenesis. Nat Commun. 2015;6:6910. 25908435 10.1038/ncomms7910 PMC4410389

[34] Liu CC , Lin JH , Hsu TW , et al. IL‐6 enriched lung cancer stem‐like cell population by inhibition of cell cycle regulators via DNMT1 upregulation. Int J Cancer. 2015;136:547‐559. 24947242 10.1002/ijc.29033

[35] Zagorac S , Alcala S , Fernandez Bayon G , et al. DNMT1 inhibition reprograms pancreatic Cancer stem cells via Upregulation of the miR‐17‐92 cluster. Cancer Res. 2016;76:4546‐4558. 27261509 10.1158/0008-5472.CAN-15-3268 PMC5295626

[36] Lee E , Wang J , Yumoto K , et al. DNMT1 regulates epithelial‐Mesenchymal transition and Cancer stem cells, which promotes prostate Cancer metastasis. Neoplasia. 2016;18:553‐566. 27659015 10.1016/j.neo.2016.07.007 PMC5031902

[37] Wajed SA , Laird PW , DeMeester TR . DNA methylation: an alternative pathway to cancer. Ann Surg. 2001;234:10‐20. 11420478 10.1097/00000658-200107000-00003 PMC1421942

[38] Ernst J , Kheradpour P , Mikkelsen TS , et al. Mapping and analysis of chromatin state dynamics in nine human cell types. Nature. 2011;473:43‐49. 21441907 10.1038/nature09906 PMC3088773

[39] Hawkins RD , Hon GC , Lee LK , et al. Distinct epigenomic landscapes of pluripotent and lineage‐committed human cells. Cell Stem Cell. 2010;6:479‐491. 20452322 10.1016/j.stem.2010.03.018 PMC2867844

[40] Pereira CF , Piccolo FM , Tsubouchi T , et al. ESCs require PRC2 to direct the successful reprogramming of differentiated cells toward pluripotency. Cell Stem Cell. 2010;6:547‐556. 20569692 10.1016/j.stem.2010.04.013

[41] Beguelin W , Popovic R , Teater M , et al. EZH2 is required for germinal center formation and somatic EZH2 mutations promote lymphoid transformation. Cancer Cell. 2013;23:677‐692. 23680150 10.1016/j.ccr.2013.04.011 PMC3681809

[42] Venkatesan N , Wong JF , Tan KP , et al. EZH2 promotes neoplastic transformation through VAV interaction‐dependent extranuclear mechanisms. Oncogene. 2018;37:461‐477. 28967906 10.1038/onc.2017.309

[43] Bracken AP , Pasini D , Capra M , Prosperini E , Colli E , Helin K . EZH2 is downstream of the pRB‐E2F pathway, essential for proliferation and amplified in cancer. EMBO J. 2003;22:5323‐5335. 14532106 10.1093/emboj/cdg542 PMC213796

[44] Kleer CG , Cao Q , Varambally S , et al. EZH2 is a marker of aggressive breast cancer and promotes neoplastic transformation of breast epithelial cells. Proc Natl Acad Sci USA. 2003;100:11606‐11611. 14500907 10.1073/pnas.1933744100 PMC208805

[45] van Vlerken LE , Kiefer CM , Morehouse C , et al. Hurt EM. EZH2 is required for breast and pancreatic cancer stem cell maintenance and can be used as a functional cancer stem cell reporter. Stem Cells Transl Med. 2013;2:43‐52. 23283488 10.5966/sctm.2012-0036 PMC3659740

[46] Arcipowski KM , Martinez CA , Ntziachristos P . Histone demethylases in physiology and cancer: a tale of two enzymes, JMJD3 and UTX. Curr Opin Genet Dev. 2016;36:59‐67. 27151432 10.1016/j.gde.2016.03.010 PMC4880520

[47] Yuan J , Takeuchi M , Negishi M , Oguro H , Ichikawa H , Iwama A . Bmi1 is essential for leukemic reprogramming of myeloid progenitor cells. Leukemia. 2011;25:1335‐1343. 21527932 10.1038/leu.2011.85

[48] Abdouh M , Facchino S , Chatoo W , Balasingam V , Ferreira J , Bernier G . BMI1 sustains human glioblastoma multiforme stem cell renewal. J Neurosci. 2009;29:8884‐8896. 19605626 10.1523/JNEUROSCI.0968-09.2009 PMC6665439

[49] Lei Y , Liu L , Zhang S , et al. Hdac7 promotes lung tumorigenesis by inhibiting Stat3 activation. Mol Cancer. 2017;16:170. 29126425 10.1186/s12943-017-0736-2 PMC5681774

[50] Mazumdar C , Shen Y , Xavy S , et al. Leukemia‐associated Cohesin mutants dominantly enforce stem cell programs and impair human hematopoietic progenitor differentiation. Cell Stem Cell. 2015;17:675‐688. 26607380 10.1016/j.stem.2015.09.017 PMC4671831

[51] Jagani Z , Mora‐Blanco EL , Sansam CG , et al. Loss of the tumor suppressor Snf5 leads to aberrant activation of the hedgehog‐Gli pathway. Nat Med. 2010;16:1429‐1433. 21076395 10.1038/nm.2251 PMC3859448

[52] Wilson BG , Roberts CW . SWI/SNF nucleosome remodellers and cancer. Nat Rev Cancer. 2011;11:481‐492. 21654818 10.1038/nrc3068

[53] Whyte WA , Orlando DA , Hnisz D , et al. Master transcription factors and mediator establish super‐enhancers at key cell identity genes. Cell. 2013;153:307‐319. 23582322 10.1016/j.cell.2013.03.035 PMC3653129

[54] Nord AS , Blow MJ , Attanasio C , et al. Rapid and pervasive changes in genome‐wide enhancer usage during mammalian development. Cell. 2013;155:1521‐1531. 24360275 10.1016/j.cell.2013.11.033 PMC3989111

[55] Herz HM . Enhancer deregulation in cancer and other diseases. Bioessays. 2016;38:1003‐1015. 27570183 10.1002/bies.201600106 PMC5160997

[56] Sur I , Taipale J . The role of enhancers in cancer. Nat Rev Cancer. 2016;16:483‐493. 27364481 10.1038/nrc.2016.62

[57] Poli V , Fagnocchi L , Fasciani A , et al. MYC‐driven epigenetic reprogramming favors the onset of tumorigenesis by inducing a stem cell‐like state. Nat Commun. 2018;9:1024. 29523784 10.1038/s41467-018-03264-2 PMC5844884

[58] Bernstein BE , Mikkelsen TS , Xie X , et al. A bivalent chromatin structure marks key developmental genes in embryonic stem cells. Cell. 2006;125:315‐326. 16630819 10.1016/j.cell.2006.02.041

[59] Bertero A , Madrigal P , Galli A , et al. Activin/nodal signaling and NANOG orchestrate human embryonic stem cell fate decisions by controlling the H3K4me3 chromatin mark. Genes Dev. 2015;29:702‐717. 25805847 10.1101/gad.255984.114 PMC4387713

[60] Suva ML , Riggi N , Bernstein BE . Epigenetic reprogramming in cancer. Science. 2013;339:1567‐1570. 23539597 10.1126/science.1230184 PMC3821556

[61] Yamazaki J , Estecio MR , Lu Y , et al. The epigenome of AML stem and progenitor cells. Epigenetics. 2013;8:92‐104. 23249680 10.4161/epi.23243 PMC3549884

[62] Zaidi SK , Frietze SE , Gordon JA , et al. Bivalent epigenetic control of Oncofetal gene expression in Cancer. Mol Cell Biol. 2017;37(23):e00352‐17. 10.1128/MCB.00352-17 PMC5686582 28923849

[63] Dunican DS , Mjoseng HK , Duthie L , Flyamer IM , Bickmore WA , Meehan RR . Bivalent promoter hypermethylation in cancer is linked to the H327me3/H3K4me3 ratio in embryonic stem cells. BMC Biol. 2020;18:25. 32131813 10.1186/s12915-020-0752-3 PMC7057567

[64] Chaffer CL , Marjanovic ND , Lee T , et al. Poised chromatin at the ZEB1 promoter enables breast cancer cell plasticity and enhances tumorigenicity. Cell. 2013;154:61‐74. 23827675 10.1016/j.cell.2013.06.005 PMC4015106

[65] Clapier CR , Cairns BR . The biology of chromatin remodeling complexes. Annu Rev Biochem. 2009;78:273‐304. 19355820 10.1146/annurev.biochem.77.062706.153223

[66] Jiang C , Pugh BF . Nucleosome positioning and gene regulation: advances through genomics. Nat Rev Genet. 2009;10:161‐172. 19204718 10.1038/nrg2522 PMC4860946

[67] Chen L , Cai Y , Jin J , et al. Subunit organization of the human INO80 chromatin remodeling complex: an evolutionarily conserved core complex catalyzes ATP‐dependent nucleosome remodeling. J Biol Chem. 2011;286:11283‐11289. 21303910 10.1074/jbc.M111.222505 PMC3064184

[68] Vignali M , Hassan AH , Neely KE , Workman JL . ATP‐dependent chromatin‐remodeling complexes. Mol Cell Biol. 2000;20:1899‐1910. 10688638 10.1128/mcb.20.6.1899-1910.2000 PMC110808

[69] Lai AY , Wade PA . Cancer biology and NuRD: a multifaceted chromatin remodelling complex. Nat Rev Cancer. 2011;11:588‐596. 21734722 10.1038/nrc3091 PMC4157524

[70] Harikrishnan KN , Chow MZ , Baker EK , et al. Brahma links the SWI/SNF chromatin‐remodeling complex with MeCP2‐dependent transcriptional silencing. Nat Genet. 2005;37:254‐264. 15696166 10.1038/ng1516

[71] Wysocka J , Swigut T , Xiao H , et al. A PHD finger of NURF couples histone H3 lysine 4 trimethylation with chromatin remodelling. Nature. 2006;442:86‐90. 16728976 10.1038/nature04815

[72] Gifford CA , Ziller MJ , Gu H , et al. Transcriptional and epigenetic dynamics during specification of human embryonic stem cells. Cell. 2013;153:1149‐1163. 23664763 10.1016/j.cell.2013.04.037 PMC3709577

[73] Chen T , Dent SY . Chromatin modifiers and remodellers: regulators of cellular differentiation. Nat Rev Genet. 2014;15:93‐106. 24366184 10.1038/nrg3607 PMC3999985

[74] Zhang W , Li Y , Kulik M , et al. Nucleosome positioning changes during human embryonic stem cell differentiation. Epigenetics. 2016;11:426‐437. 27088311 10.1080/15592294.2016.1176649 PMC4939925

[75] Gao X , Tate P , Hu P , Tjian R , Skarnes WC , Wang Z . ES cell pluripotency and germ‐layer formation require the SWI/SNF chromatin remodeling component BAF250a. Proc Natl Acad Sci USA. 2008;105:6656‐6661. 18448678 10.1073/pnas.0801802105 PMC2373334

[76] West JA , Cook A , Alver BH , et al. Nucleosomal occupancy changes locally over key regulatory regions during cell differentiation and reprogramming. Nat Commun. 2014;5:4719. 25158628 10.1038/ncomms5719 PMC4217530

[77] Neely KE , Workman JL . The complexity of chromatin remodeling and its links to cancer. Biochim Biophys Acta. 2002;1603:19‐29. 12242108 10.1016/s0304-419x(02)00067-7

[78] Shain AH , Pollack JR . The spectrum of SWI/SNF mutations, ubiquitous in human cancers. PLoS One. 2013;8:e55119. 23355908 10.1371/journal.pone.0055119 PMC3552954

[79] Oike T , Ogiwara H , Nakano T , Yokota J , Kohno T . Inactivating mutations in SWI/SNF chromatin remodeling genes in human cancer. Jpn J Clin Oncol. 2013;43:849‐855. 23904343 10.1093/jjco/hyt101

[80] Buscarlet M , Krasteva V , Ho L , et al. Essential role of BRG, the ATPase subunit of BAF chromatin remodeling complexes, in leukemia maintenance. Blood. 2014;123:1720‐1728. 24478402 10.1182/blood-2013-02-483495 PMC3954053

[81] Shi J , Whyte WA , Zepeda‐Mendoza CJ , et al. Role of SWI/SNF in acute leukemia maintenance and enhancer‐mediated Myc regulation. Genes Dev. 2013;27:2648‐2662. 24285714 10.1101/gad.232710.113 PMC3877755

[82] Marquez‐Vilendrer SB , Rai SK , Gramling SJ , Lu L , Reisman DN . Loss of the SWI/SNF ATPase subunits BRM and BRG1 drives lung cancer development. Onco Targets Ther. 2016;3:322‐336. 10.18632/oncoscience.323 PMC5235921 28105457

[83] Chai J , Charboneau AL , Betz BL , Weissman BE . Loss of the hSNF5 gene concomitantly inactivates p21CIP/WAF1 and p16INK4a activity associated with replicative senescence in A204 rhabdoid tumor cells. Cancer Res. 2005;65:10192‐10198. 16288006 10.1158/0008-5472.CAN-05-1896

[84] McKenna ES , Roberts CW . Epigenetics and cancer without genomic instability. Cell Cycle. 2009;8:23‐26. 19098432 10.4161/cc.8.1.7290

[85] McKenna ES , Sansam CG , Cho YJ , et al. Loss of the epigenetic tumor suppressor SNF5 leads to cancer without genomic instability. Mol Cell Biol. 2008;28:6223‐6233. 18710953 10.1128/MCB.00658-08 PMC2577431

[86] Sarma K , Reinberg D . Histone variants meet their match. Nat Rev Mol Cell Biol. 2005;6:139‐149. 15688000 10.1038/nrm1567

[87] Jin C , Zang C , Wei G , et al. H3.3/H2A.Z double variant‐containing nucleosomes mark ‘nucleosome‐free regions’ of active promoters and other regulatory regions. Nat Genet. 2009;41:941‐945. 19633671 10.1038/ng.409 PMC3125718

[88] Chakravarthy S , Luger K . The histone variant macro‐H2A preferentially forms "hybrid nucleosomes". J Biol Chem. 2006;281:25522‐25531. 16803903 10.1074/jbc.M602258200

[89] Boskovic A , Eid A , Pontabry J , et al. Higher chromatin mobility supports totipotency and precedes pluripotency in vivo. Genes Dev. 2014;28:1042‐1047. 24831699 10.1101/gad.238881.114 PMC4035533

[90] Santenard A , Torres‐Padilla ME . Epigenetic reprogramming in mammalian reproduction: contribution from histone variants. Epigenetics. 2009;4:80‐84. 19242119 10.4161/epi.4.2.7838

[91] Turinetto V , Giachino C . Histone variants as emerging regulators of embryonic stem cell identity. Epigenetics. 2015;10:563‐573. 26114724 10.1080/15592294.2015.1053682 PMC4622597

[92] Creppe C , Janich P , Cantarino N , et al. MacroH2A1 regulates the balance between self‐renewal and differentiation commitment in embryonic and adult stem cells. Mol Cell Biol. 2012;32:1442‐1452. 22331466 10.1128/MCB.06323-11 PMC3318583

[93] Kapoor A , Goldberg MS , Cumberland LK , et al. The histone variant macroH2A suppresses melanoma progression through regulation of CDK8. Nature. 2010;468:1105‐1109. 21179167 10.1038/nature09590 PMC3057940

[94] Lo Re O , Fusilli C , Rappa F , et al. Induction of cancer cell stemness by depletion of macrohistone H2A1 in hepatocellular carcinoma. Hepatology. 2018;67:636‐650. 28913935 10.1002/hep.29519

[95] Park SJ , Shim JW , Park HS , et al. MacroH2A1 downregulation enhances the stem‐like properties of bladder cancer cells by transactivation of Lin28B. Oncogene. 2016;35:1292‐1301. 26028027 10.1038/onc.2015.187 PMC4791524

[96] Zink LM , Hake SB . Histone variants: nuclear function and disease. Curr Opin Genet Dev. 2016;37:82‐89. 26826795 10.1016/j.gde.2015.12.002

[97] Hua S , Kallen CB , Dhar R , et al. Genomic analysis of estrogen cascade reveals histone variant H2A.Z associated with breast cancer progression. Mol Syst Biol. 2008;4:188. 18414489 10.1038/msb.2008.25 PMC2394496

[98] Vardabasso C , Gaspar‐Maia A , Hasson D , et al. Histone variant H2A.Z.2 mediates proliferation and drug sensitivity of malignant melanoma. Mol Cell. 2015;59:75‐88. 26051178 10.1016/j.molcel.2015.05.009 PMC4490946

[99] Yang HD , Kim PJ , Eun JW , et al. Oncogenic potential of histone‐variant H2A.Z.1 and its regulatory role in cell cycle and epithelial‐mesenchymal transition in liver cancer. Oncotarget. 2016;7:11412‐11423. 26863632 10.18632/oncotarget.7194 PMC4905482

[100] Gallo M , Coutinho FJ , Vanner RJ , et al. MLL5 orchestrates a Cancer self‐renewal state by repressing the histone variant H3.3 and globally reorganizing chromatin. Cancer Cell. 2015;28:715‐729. 26626085 10.1016/j.ccell.2015.10.005

[101] Esteller M . Non‐coding RNAs in human disease. Nat Rev Genet. 2011;12:861‐874. 22094949 10.1038/nrg3074

[102] Wang KC , Chang HY . Molecular mechanisms of long noncoding RNAs. Mol Cell. 2011;43:904‐914. 21925379 10.1016/j.molcel.2011.08.018 PMC3199020

[103] Guttman M , Donaghey J , Carey BW , et al. lincRNAs act in the circuitry controlling pluripotency and differentiation. Nature. 2011;477:295‐300. 21874018 10.1038/nature10398 PMC3175327

[104] Kim DH , Marinov GK , Pepke S , et al. Single‐cell transcriptome analysis reveals dynamic changes in lncRNA expression during reprogramming. Cell Stem Cell. 2015;16:88‐101. 25575081 10.1016/j.stem.2014.11.005 PMC4291542

[105] Huang Y , Zheng Y , Jin C , Li X , Jia L , Li W . Long non‐coding RNA H19 inhibits adipocyte differentiation of bone marrow Mesenchymal stem cells through epigenetic modulation of histone Deacetylases. Sci Rep. 2016;6:28897. 27349231 10.1038/srep28897 PMC4924093

[106] Jiang X , Yan Y , Hu M , et al. Increased level of H19 long noncoding RNA promotes invasion, angiogenesis, and stemness of glioblastoma cells. J Neurosurg. 2016;2016:129‐136. 28306408 10.3171/2014.12.JNS1426.test

[107] Loewer S , Cabili MN , Guttman M , et al. Large intergenic non‐coding RNA‐RoR modulates reprogramming of human induced pluripotent stem cells. Nat Genet. 2010;42:1113‐1117. 21057500 10.1038/ng.710 PMC3040650

[108] Gao S , Wang P , Hua Y , et al. ROR functions as a ceRNA to regulate Nanog expression by sponging miR‐145 and predicts poor prognosis in pancreatic cancer. Oncotarget. 2016;7:1608‐1618. 26636540 10.18632/oncotarget.6450 PMC4811484

[109] Lou Y , Jiang H , Cui Z , Wang L , Wang X , Tian T . Linc‐ROR induces epithelial‐to‐mesenchymal transition in ovarian cancer by increasing Wnt/beta‐catenin signaling. Oncotarget. 2017;8:69983‐69994. 29050257 10.18632/oncotarget.19545 PMC5642532

[110] Peng WX , Huang JG , Yang L , Gong AH , Mo YY . Linc‐RoR promotes MAPK/ERK signaling and confers estrogen‐independent growth of breast cancer. Mol Cancer. 2017;16:161. 29041978 10.1186/s12943-017-0727-3 PMC5645922

[111] Rezaei M , Emadi‐Baygi M , Hoffmann MJ , Schulz WA , Nikpour P . Altered expression of LINC‐ROR in cancer cell lines and tissues. Tumour Biol. 2016;37:1763‐1769. 26314857 10.1007/s13277-015-3933-x

[112] Wang L , Yu X , Zhang Z , et al. Linc‐ROR promotes esophageal squamous cell carcinoma progression through the derepression of SOX9. J Exp Clin Cancer Res. 2017;36:182. 29237490 10.1186/s13046-017-0658-2 PMC5727696

[113] Zhang R , Hardin H , Huang W , Buehler D , Lloyd RV . Long non‐coding RNA Linc‐ROR is Upregulated in papillary thyroid carcinoma. Endocr Pathol. 2018;29:1‐8. 29280051 10.1007/s12022-017-9507-2

[114] Zhan HX , Wang Y , Li C , et al. LincRNA‐ROR promotes invasion, metastasis and tumor growth in pancreatic cancer through activating ZEB1 pathway. Cancer Lett. 2016;374:261‐271. 26898939 10.1016/j.canlet.2016.02.018

[115] Gupta RA , Shah N , Wang KC , et al. Long non‐coding RNA HOTAIR reprograms chromatin state to promote cancer metastasis. Nature. 2010;464:1071‐1076. 20393566 10.1038/nature08975 PMC3049919

[116] Padua Alves C , Fonseca AS , Muys BR , et al. Brief report: the lincRNA Hotair is required for epithelial‐to‐mesenchymal transition and stemness maintenance of cancer cell lines. Stem Cells. 2013;31:2827‐2832. 24022994 10.1002/stem.1547

[117] Iorio MV , Croce CM . MicroRNA dysregulation in cancer: diagnostics, monitoring and therapeutics. A comprehensive review. EMBO Mol Med. 2012;4:143‐159. 22351564 10.1002/emmm.201100209 PMC3376845

[118] Bernstein E , Kim SY , Carmell MA , et al. Dicer is essential for mouse development. Nat Genet. 2003;35:215‐217. 14528307 10.1038/ng1253

[119] Wang Y , Medvid R , Melton C , Jaenisch R , Blelloch R . DGCR8 is essential for microRNA biogenesis and silencing of embryonic stem cell self‐renewal. Nat Genet. 2007;39:380‐385. 17259983 10.1038/ng1969 PMC3008549

[120] Melton C , Judson RL , Blelloch R . Opposing microRNA families regulate self‐renewal in mouse embryonic stem cells. Nature. 2010;463:621‐626. 20054295 10.1038/nature08725 PMC2894702

[121] Viswanathan SR , Daley GQ , Gregory RI . Selective blockade of microRNA processing by Lin28. Science. 2008;320:97‐100. 18292307 10.1126/science.1154040 PMC3368499

[122] Wang L , Su Y , Huang C , et al. NANOG and LIN28 dramatically improve human cell reprogramming by modulating LIN41 and canonical WNT activities. Biol Open. 2019;8:bio047225. 31806618 10.1242/bio.047225 PMC6918770

[123] Yu F , Yao H , Zhu P , et al. Let‐7 regulates self renewal and tumorigenicity of breast cancer cells. Cell. 2007;131:1109‐1123. 18083101 10.1016/j.cell.2007.10.054

[124] Albino D , Civenni G , Dallavalle C , et al. Activation of the Lin28/let‐7 Axis by loss of ESE3/EHF promotes a tumorigenic and stem‐like phenotype in prostate Cancer. Cancer Res. 2016;76:3629‐3643. 27197175 10.1158/0008-5472.CAN-15-2665

[125] Guo L , Cheng X , Chen H , et al. Induction of breast cancer stem cells by M1 macrophages through Lin‐28B‐let‐7‐HMGA2 axis. Cancer Lett. 2019;452:213‐225. 30917918 10.1016/j.canlet.2019.03.032

[126] Ottaviani S , Castellano L . microRNAs: novel regulators of the TGF‐beta pathway in pancreatic ductal adenocarcinoma. Mol Cell Oncol. 2018;5:e1499066. 30525087 10.1080/23723556.2018.1499066 PMC6276843

[127] Yang X , Lin X , Zhong X , et al. Double‐negative feedback loop between reprogramming factor LIN28 and microRNA let‐7 regulates aldehyde dehydrogenase 1‐positive cancer stem cells. Cancer Res. 2010;70:9463‐9472. 21045151 10.1158/0008-5472.CAN-10-2388 PMC3057570

[128] Iliopoulos D , Hirsch HA , Struhl K . An epigenetic switch involving NF‐kappaB, Lin28, Let‐7 MicroRNA, and IL6 links inflammation to cell transformation. Cell. 2009;139:693‐706. 19878981 10.1016/j.cell.2009.10.014 PMC2783826

[129] Viswanathan SR , Powers JT , Einhorn W , et al. Lin28 promotes transformation and is associated with advanced human malignancies. Nat Genet. 2009;41:843‐848. 19483683 10.1038/ng.392 PMC2757943

[130] Wang YC , Chen YL , Yuan RH , et al. Lin‐28B expression promotes transformation and invasion in human hepatocellular carcinoma. Carcinogenesis. 2010;31:1516‐1522. 20525879 10.1093/carcin/bgq107

[131] Davis BN , Hilyard AC , Lagna G , Hata A . SMAD proteins control DROSHA‐mediated microRNA maturation. Nature. 2008;454:56‐61. 18548003 10.1038/nature07086 PMC2653422

[132] Asangani IA , Rasheed SA , Nikolova DA , et al. MicroRNA‐21 (miR‐21) post‐transcriptionally downregulates tumor suppressor Pdcd4 and stimulates invasion, intravasation and metastasis in colorectal cancer. Oncogene. 2008;27:2128‐2136. 17968323 10.1038/sj.onc.1210856

[133] Song SJ , Poliseno L , Song MS , et al. MicroRNA‐antagonism regulates breast cancer stemness and metastasis via TET‐family‐dependent chromatin remodeling. Cell. 2013;154:311‐324. 23830207 10.1016/j.cell.2013.06.026 PMC3767157

[134] Iliou MS , Lujambio A , Portela A , et al. Bivalent histone modifications in stem cells poise miRNA loci for CpG Island hypermethylation in human cancer. Epigenetics. 2011;6:1344‐1353. 22048248 10.4161/epi.6.11.18021

[135] Mani SA , Guo W , Liao MJ , et al. The epithelial‐mesenchymal transition generates cells with properties of stem cells. Cell. 2008;133:704‐715. 18485877 10.1016/j.cell.2008.03.027 PMC2728032

[136] Morel AP , Lievre M , Thomas C , Hinkal G , Ansieau S , Puisieux A . Generation of breast cancer stem cells through epithelial‐mesenchymal transition. PLoS One. 2008;3:e2888. 18682804 10.1371/journal.pone.0002888 PMC2492808

[137] Ocana OH , Corcoles R , Fabra A , et al. Metastatic colonization requires the repression of the epithelial‐mesenchymal transition inducer Prrx1. Cancer Cell. 2012;22:709‐724. 23201163 10.1016/j.ccr.2012.10.012

[138] Chang CJ , Chao CH , Xia W , et al. p53 regulates epithelial‐mesenchymal transition and stem cell properties through modulating miRNAs. Nat Cell Biol. 2011;13:317‐323. 21336307 10.1038/ncb2173 PMC3075845

[139] Wan S , Zhao E , Kryczek I , et al. Tumor‐associated macrophages produce interleukin 6 and signal via STAT3 to promote expansion of human hepatocellular carcinoma stem cells. Gastroenterology. 2014;147:1393‐1404. 25181692 10.1053/j.gastro.2014.08.039 PMC4253315

[140] Nakano M , Kikushige Y , Miyawaki K , et al. Dedifferentiation process driven by TGF‐beta signaling enhances stem cell properties in human colorectal cancer. Oncogene. 2019;38:780‐793. 30181548 10.1038/s41388-018-0480-0

[141] Cui TX , Kryczek I , Zhao L , et al. Myeloid‐derived suppressor cells enhance stemness of cancer cells by inducing microRNA101 and suppressing the corepressor CtBP2. Immunity. 2013;39:611‐621. 24012420 10.1016/j.immuni.2013.08.025 PMC3831370

[142] Peng D , Tanikawa T , Li W , et al. Myeloid‐derived suppressor cells endow stem‐like qualities to breast Cancer cells through IL6/STAT3 and NO/NOTCH cross‐talk signaling. Cancer Res. 2016;76:3156‐3165. 27197152 10.1158/0008-5472.CAN-15-2528 PMC4891237

[143] Sultan M , Vidovic D , Paine AS , et al. Epigenetic silencing of TAP1 in Aldefluor(+) breast Cancer stem cells contributes to their enhanced immune evasion. Stem Cells. 2018;36:641‐654. 29341428 10.1002/stem.2780

[144] Diaz‐Lagares A , Mendez‐Gonzalez J , Hervas D , et al. A novel epigenetic signature for early diagnosis in lung cancer. Clin Cancer Res. 2016;22:3361‐3371. 26842235 10.1158/1078-0432.CCR-15-2346

[145] McAnena P , Brown JA , Kerin MJ . Circulating nucleosomes and nucleosome modifications as biomarkers in cancer. Cancers (Basel). 2017;9(1):5. 10.3390/cancers9010005 PMC5295776 28075351

[146] Murtaza M , Caldas C . Nucleosome mapping in plasma DNA predicts cancer gene expression. Nat Genet. 2016;48:1105‐1106. 27681289 10.1038/ng.3686

[147] Bolha L , Ravnik‐Glavac M , Glavac D . Circular RNAs: biogenesis, function, and a role as possible Cancer biomarkers. Int J Genomics. 2017;2017:6218353. 29349062 10.1155/2017/6218353 PMC5733622

[148] Filipow S , Laczmanski L . Blood circulating miRNAs as Cancer biomarkers for diagnosis and surgical treatment response. Front Genet. 2019;10:169. 30915102 10.3389/fgene.2019.00169 PMC6421259

[149] Chen WX , Hu Q , Qiu MT , et al. miR‐221/222: promising biomarkers for breast cancer. Tumour Biol. 2013;34:1361‐1370. 23529451 10.1007/s13277-013-0750-y

[150] Kawaguchi T , Komatsu S , Ichikawa D , et al. Clinical impact of circulating miR‐221 in plasma of patients with pancreatic cancer. Br J Cancer. 2013;108:361‐369. 23329235 10.1038/bjc.2012.546 PMC3566805

[151] Zhang H , Mao F , Shen T , et al. Plasma miR‐145, miR‐20a, miR‐21 and miR‐223 as novel biomarkers for screening early‐stage non‐small cell lung cancer. Oncol Lett. 2017;13:669‐676. 28356944 10.3892/ol.2016.5462 PMC5351202

[152] Shin VY , Siu JM , Cheuk I , Ng EK , Kwong A . Circulating cell‐free miRNAs as biomarker for triple‐negative breast cancer. Br J Cancer. 2015;112:1751‐1759. 25906045 10.1038/bjc.2015.143 PMC4647231

[153] Roscigno G , Quintavalle C , Donnarumma E , et al. MiR‐221 promotes stemness of breast cancer cells by targeting DNMT3b. Oncotarget. 2016;7:580‐592. 26556862 10.18632/oncotarget.5979 PMC4808019

[154] Mamoori A , Gopalan V , Smith RA , Lam AK . Modulatory roles of microRNAs in the regulation of different signalling pathways in large bowel cancer stem cells. Biol Cell. 2016;108:51‐64. 26712035 10.1111/boc.201500062

[155] Sekar D , Krishnan R , Panagal M , Sivakumar P , Gopinath V , Basam V . Deciphering the role of microRNA 21 in cancer stem cells (CSCs). Genes Dis. 2016;3:277‐281. 30258897 10.1016/j.gendis.2016.05.002 PMC6147178

[156] Eckschlager T , Plch J , Stiborova M , Hrabeta J . Histone Deacetylase inhibitors as anticancer drugs. Int J Mol Sci. 2017;18:1414. 10.3390/ijms18071414 PMC5535906 28671573

[157] Goldstein LJ , Zhao F , Wang M , et al. A phase I/II study of suberoylanilide hydroxamic acid (SAHA) in combination with trastuzumab (Herceptin) in patients with advanced metastatic and/or local chest wall recurrent HER2‐amplified breast cancer: a trial of the ECOG‐ACRIN Cancer research group (E1104). Breast Cancer Res Treat. 2017;165:375‐382. 28623430 10.1007/s10549-017-4310-9 PMC5682621

[158] Subramanian S , Bates SE , Wright JJ , Espinoza‐Delgado I , Piekarz RL . Clinical toxicities of histone Deacetylase inhibitors. Pharmaceuticals (Basel). 2010;3:2751‐2767. 27713375 10.3390/ph3092751 PMC4034096

[159] Witt AE , Lee CW , Lee TI , et al. Identification of a cancer stem cell‐specific function for the histone deacetylases, HDAC1 and HDAC7, in breast and ovarian cancer. Oncogene. 2017;36:1707‐1720. 27694895 10.1038/onc.2016.337 PMC5364039

[160] Debeb BG , Lacerda L , Xu W , et al. Histone deacetylase inhibitors stimulate dedifferentiation of human breast cancer cells through WNT/beta‐catenin signaling. Stem Cells. 2012;30:2366‐2377. 22961641 10.1002/stem.1219 PMC4545658

[161] Filippakopoulos P , Qi J , Picaud S , et al. Selective inhibition of BET bromodomains. Nature. 2010;468:1067‐1073. 20871596 10.1038/nature09504 PMC3010259

[162] Leal AS , Williams CR , Royce DB , Pioli PA , Sporn MB , Liby KT . Bromodomain inhibitors, JQ1 and I‐BET 762, as potential therapies for pancreatic cancer. Cancer Lett. 2017;394:76‐87. 28254412 10.1016/j.canlet.2017.02.021

[163] Mazur PK , Herner A , Mello SS , et al. Combined inhibition of BET family proteins and histone deacetylases as a potential epigenetics‐based therapy for pancreatic ductal adenocarcinoma. Nat Med. 2015;21:1163‐1171. 26390243 10.1038/nm.3952 PMC4959788

[164] Wen N , Guo B , Zheng H , et al. Bromodomain inhibitor jq1 induces cell cycle arrest and apoptosis of glioma stem cells through the VEGF/PI3K/AKT signaling pathway. Int J Oncol. 2019;55:879‐895. 31485609 10.3892/ijo.2019.4863 PMC6741838

[165] Wang J , Hevi S , Kurash JK , et al. The lysine demethylase LSD1 (KDM1) is required for maintenance of global DNA methylation. Nat Genet. 2009;41:125‐129. 19098913 10.1038/ng.268

[166] Pedersen MT , Helin K . Histone demethylases in development and disease. Trends Cell Biol. 2010;20:662‐671. 20863703 10.1016/j.tcb.2010.08.011

[167] Ueda R , Suzuki T , Mino K , et al. Identification of cell‐active lysine specific demethylase 1‐selective inhibitors. J Am Chem Soc. 2009;131:17536‐17537. 19950987 10.1021/ja907055q

[168] Sareddy GR , Viswanadhapalli S , Surapaneni P , Suzuki T , Brenner A , Vadlamudi RK . Novel KDM1A inhibitors induce differentiation and apoptosis of glioma stem cells via unfolded protein response pathway. Oncogene. 2017;36:2423‐2434. 27893719 10.1038/onc.2016.395 PMC5526658

[169] Jin M , Zhang T , Liu C , et al. miRNA‐128 suppresses prostate cancer by inhibiting BMI‐1 to inhibit tumor‐initiating cells. Cancer Res. 2014;74:4183‐4195. 24903149 10.1158/0008-5472.CAN-14-0404 PMC4174451

[170] Wang Y , Zhe H , Ding Z , Gao P , Zhang N , Li G . Cancer stem cell marker Bmi‐1 expression is associated with basal‐like phenotype and poor survival in breast cancer. World J Surg. 2012;36:1189‐1194. 22366984 10.1007/s00268-012-1514-3

[171] Kreso A , van Galen P , Pedley NM , et al. Self‐renewal as a therapeutic target in human colorectal cancer. Nat Med. 2014;20:29‐36. 24292392 10.1038/nm.3418

[172] Gan L , Xu M , Hua R , et al. The polycomb group protein EZH2 induces epithelial‐mesenchymal transition and pluripotent phenotype of gastric cancer cells by binding to PTEN promoter. J Hematol Oncol. 2018;11:9. 29335012 10.1186/s13045-017-0547-3 PMC5769437

[173] Yu T , Wang Y , Hu Q , et al. The EZH2 inhibitor GSK343 suppresses cancer stem‐like phenotypes and reverses mesenchymal transition in glioma cells. Oncotarget. 2017;8:98348‐98359. 29228694 10.18632/oncotarget.21311 PMC5716734

[174] Roos M , Pradere U , Ngondo RP , et al. A small‐molecule inhibitor of Lin28. ACS Chem Biol. 2016;11:2773‐2781. 27548809 10.1021/acschembio.6b00232

[175] Ji W , Sun B , Su C . Targeting MicroRNAs in Cancer gene therapy. Genes (Basel). 2017;8(1):21. 10.3390/genes8010021 PMC5295016 28075356

[176] Montazersaheb S , Hejazi MS , Nozad Charoudeh H . Potential of peptide nucleic acids in future therapeutic applications. Adv Pharm Bull. 2018;8:551‐563. 30607328 10.15171/apb.2018.064 PMC6311635

[177] Ozes AR , Wang Y , Zong X , Fang F , Pilrose J , Nephew KP . Therapeutic targeting using tumor specific peptides inhibits long non‐coding RNA HOTAIR activity in ovarian and breast cancer. Sci Rep. 2017;7:894. 28420874 10.1038/s41598-017-00966-3 PMC5429858

